# Bereaved Parents as Communication Workshop Facilitators for Clinicians Caring for Seriously Ill Children

**DOI:** 10.1089/pmr.2025.0022

**Published:** 2025-06-09

**Authors:** Camara Van Breemen, Nadine Lusney, Anne-Mette Hermansen, Lee Vang

**Affiliations:** ^1^ Canuck Place Children’s Hospice, Vancouver, Canada.; ^2^ Faculty of Medicine, University of British Columbia, Vancouver, Canada.; ^3^ BC Children’s Hospital, Vancouver, Canada.; ^4^ BC Children’s Hospital Research Institute, Vancouver, Canada.; ^5^ Family Faculty, Education Program, Canuck Place Children’s Hospice.

**Keywords:** advance care planning, communication training, education, family engagement, illness conversation, pediatric palliative care, serious

## Abstract

**Introduction:**

The Serious Illness Conversation Guide-Pediatrics (SICG-Peds) is a validated tool and training program that increases clinicians’ confidence in leading complex conversations with seriously ill pediatric patients and their families. We initiated a pilot project incorporating bereaved parents as facilitators in SICG-Peds education.

**Objectives:**

To assess how incorporating bereaved parents in a facilitator role in the SICG-Peds education program impacted the experience for clinician trainees and clinical facilitators and the parents themselves.

**Methods:**

Four bereaved parents were onboarded and included as family facilitators. Workshop experience was measured through post-workshop surveys. Clinical facilitators and family facilitators provided feedback about the co-teaching experience.

**Results:**

Clinicians reported that having bereaved parents as teaching faculty enriched their learning. Clinical facilitators found that family facilitators offered additional perspectives and value. Parents recognized that they could hone their story and experience to support the clinician learners in unique ways.

**Conclusions:**

The addition of family facilitators in the delivery of SICG-Peds workshops enhanced clinicians’ learning. Moreover, bereaved parents reported that functioning as workshop facilitators was a deeply meaningful experience.

## Introduction 

Increasingly, patient perspectives are seen as necessary at all levels of health care, including clinical care, health research, and health resource allocation.^1,2^ Canuck Place Children’s Hospice (CPCH) in Vancouver, Canada, values patient partner participation in program development, organizational governance, and a variety of education opportunities.

Since 2018, CPCH has been using an adaption of the Serious Illness Conversation Guide (SICG) developed by Ariadne Labs.^3,4^ The SICG-Peds tool is a clinician-led guide to explore parents’ understanding of their child’s illness, discern family preferences around how to receive information, provide information about what may be ahead, and gain insight into the family’s goals, fears, strengths, and priorities in the care of their child.

The SICG-Peds program and evaluation of the implementation of SICG-Peds have been thoroughly described in Doherty et al. 2023 and van Breemen et. al 2024.^5,6^ The evaluation showed that this tool and training do increase clinician’s overall confidence in exploring parent’s values, goals, and priorities in their child’s care, as evidenced by postworkshop surveys and clinician interviews.

In 2022, we developed a program and training module for CPCH bereaved family members to become SICG-Peds family facilitators. We report the results of this pilot program.

## Methods

An email outlining the role of Family Facilitator within the SICG-Peds training was sent to >120 bereaved family members affiliated with a CPCH program who had a death of a child between 2010 and 2020. Eighteen family members (17 mothers and 1 adult sibling) sought out the opportunity to learn more about the project through a virtual appointment with the project leads. All interested family members were screened, and their intent to participate was explored in a 30–60-minute semi-structured Zoom meeting. The selected family facilitators and project leads reviewed the engagement commitments, compensation, and signed a memorandum of understanding together. The other interested partners were also provided with a follow-up phone call to thank them for their interest and provide feedback regarding the selection process.

Initial education, training, and onboarding of the family facilitators included a series of meetings with the project leads to explore best communication practices and to develop competencies on adult learning principles and application of lived experience within the context of the training. Family facilitators also completed the SICG-peds training together with the project leads and experienced the clinician role, parent actor role, and observer, along with the practice of debriefing clinician learners and the specific role of family facilitator. In addition to the workshop debriefing that occurs after each training for all facilitators, the family facilitators receive additional consolidation mentoring through as-needed check-ins, quarterly group meetings with training leads, parent-to-parent reconnects, and email communication. Family facilitators perceived their time together as vital to developing a sense of collective agreement on the work and self-determination in their roles as family facilitators. In addition, family facilitators kept reflective journals to document their experience, and project leads provided journal prompts along their journey. Journals were thematically analyzed by the project team, and themes were discussed with family facilitators to affirm understanding and provide opportunity to evolve. The family facilitators engaged in training sessions virtually based on their availability and were compensated for the hours of work. This was illustrated by a family facilitator’s reflection on the process as, “Overall, the group reconnects, the journaling, and the training debriefs all have supported me to hone my voice in my role as a family faculty member. I feel I am more articulate in sharing my family’s experience of caring for our child and how we lived through her illness and death. I am also able to be more precise in expressing my belief that good communication between medical professionals and families is critical during a child’s serious illness.” In addition, the family facilitators participated in interviews for the development of a video that explores the purpose of serious illness conversations from a family lens. This is now used in all the SICG-Peds workshops offered to clinicians.

A postworkshop survey was used to evaluate the experience of clinician learners, clinician facilitators, and family facilitators. The following question was added to the postworkshop survey for clinician learners to assess the impact of including family facilitators: “*Did having the perspective of a family partner participant support your learning during the workshop?”.* Family facilitators were also asked the following question in postworkshop surveys: “*Overall, how would you rate this experience as a Family Partner Faculty?”* Descriptive statistics of professional backgrounds and survey answers were computed in R (V4.4.1) and RStudio (V2024.09.1 + 394). Surveys were evaluated from September 2022 to June 2023 for consideration during the first year of the pilot.

This pilot project was approved by the senior leadership team and reviewed by the Quality Council at Canuck Place. No institutional ethics review was required for this quality improvement project.

## Results

Four bereaved mothers were selected for the training, based on individual strengths in communication, the ability to share lived experience, and the diversity of their health care journey (diagnosis, age of child, location of death, and length of illness). After onboarding and training, family facilitators started participating in SICG-Peds workshops in September 2022. To date, one or more family facilitator has participated in 28 SICG-Peds workshops as a co-facilitators.

Between September 2022 and June 2023, 49 health professionals participated in a family facilitator-guided workshop and provided valid responses to the postworkshop survey. Of these, 21 (43%) were physicians/nurse practitioners/fellows, 17 (35%) were registered nurses/advanced practice nurses, 3 (6%) were family engagement advisors, and 8 (16%) represented allied health professions. Participants had varying levels of professional experience, ranging from less than 1 year to over 15 years, with the majority, 34 (69%), having 0–5 years of experience. All participants (100%, *N* = 49) and clinician faculty (100%, *N* = 5) who responded to the postworkshop survey reported a positive response to the inclusion of a family partner in the workshop.

One participant shared, “Having parents’ perspectives is extremely valuable as it helps sensitize clinicians to the needs and struggles a family experiences when faced with a serious illness diagnosis. It makes it more “real” and highlights the importance of being mindful and caring when having these conversations.”

Over the same period, family facilitators participated in postworkshop surveys on 13 occasions. They rated all their workshop experiences very positively. Quotes from their surveys recognized their broadened perspective of the clinician and allowed them to enhance the learning environment by providing insights into language and relational practice. One family facilitator noted, “The more I partake in these trainings, the more skill I feel I have. My lived experience is rich with knowledge and wisdom gained over the years. To be able to hone it, use it with restraint and clarity, and help guide clinicians is a phenomenal feeling.” 100% had received “Very Good” to “Excellent” evaluations from the trainees. Self-reflective journal entries illustrated how family facilitators gained confidence and knowledge over time. One family facilitator described this as “an evolving process of sharing my story, listening and observing how it helps the learners/clinicians, and then honing my story to be more reflective of the learning needs.” Journal entries of family facilitators reflected on the importance of language and clinician embodied listening as “if words could move, they’d reach across the table, grasp the hands of the parent, cold with dread and fear, and slowly warm them, letting them thaw and slowly grasp their new reality.”

Project leads who proposed this model recognized that the role of family facilitator could have wide-reaching effects that not only enhanced communication training, but also supported grief work and meaning-making for parents. The journaling by family facilitators showed how the participation in workshops provided opportunities to have ongoing connection with their child and their grief journey. In addition, clinician facilitators reported unanimously that they believed the presence of family facilitators impacted the learning of clinicians positively. Clinician facilitators detailed concrete ways in which family facilitators may have impacted clinician practice and how the family facilitator’s presence provided opportunities for all to learn and fine-tune communication skills, i.e., by providing a different perspective on the language used in communication with families and adding depth to the relational human-ness of practice and teaching.

## Discussion

The addition of family facilitators in the SICG-Peds education program is a living example of the initiative for the pediatric palliative care model for involving families as educators.^7,8^ Weaving in the parents’ lived experience and real-life examples augmented the learning environment and promoted mutual learning. In addition, our initiative illustrated positive impacts on family facilitators such as empowerment and increased self-confidence in their teaching skills. This is similar to unintended findings from other family faculty education programs.^8^ Clinicians learn firsthand how their communication can help, hinder, or harm parents both in the short- and longer-term. Family facilitators provided concrete examples of how clinicians communicated with them along the illness trajectory. They also discussed important aspects of communication in settings in which their child was cared for, such as at times of crisis, when being discharged from the hospital, at home visits, or when transitioning to the hospice. These stories expanded upon the questions in the guide to enhance clinicians’ understanding of the importance of the situations and environments where good communication practices are key. This is motivating and builds an understanding in both novice and seasoned clinicians to consider more deeply how they approach these difficult conversations and the importance of providing guidance. This integration of family voice, lived experience, and humanness into education integrates the necessary experiential sources of knowing (both clinician and parent) with cognitive understanding to facilitate relational learning as proposed by Browning et al.^9^ Family facilitators participated in discussions as well as in the role-play debriefing. The clinician facilitator and family facilitator in the debrief offer their feedback on how the clinician may augment their communication practices, promoting “connecting” language. Common feedback includes exploring better ways to use tone, pacing, and body language.

Although the concept of having family voice present in education is not new and is now more widely accepted as a standard, questions about working with bereaved parents are important to address, both at the individual and organizational level. Will an initiative like the one we have described here cause any harm to bereaved parents engaged as family facilitators and how do we mitigate any such risk? It goes beyond the purpose of this article to answer those questions; however, the project leads took several steps to address them in their training and work with family facilitators. That included citing relevant research on patient engagement, developing a clear set of agreements with the family facilitators regarding roles and responsibilities, and outlining a pathway to receive support if needed. Most importantly, family facilitators were encouraged to give direct feedback on their experience and share positive and negative aspects of their journey. It is clear from their perspective that the engagement has made a positive impact in their lives.

This pilot project was a quality improvement/proof of concept design; thus, the rigor of the analysis lacked application to research. The project and characteristics of the family facilitator role were difficult to define in the recruitment process, and this may have discouraged some who wanted to apply or discuss further. In addition, recruitment strategies required English speaking and health literacy; thus, socioeconomic and cultural diversity was not represented. It is difficult to know how best to recruit and widen the diversity of those who would like to engage while also supporting the needs of complex skills in communication.

## Conclusion

Workshop participants are overwhelmingly positive regarding the addition of family facilitators, stating that their presence brings depth and breadth to the workshop. Family facilitators have expressed that they feel they are honoring their child by participating. Ultimately, they hope that their involvement will improve clinicians’ ability to communicate and encourage engagement in difficult conversations, a legacy that will make the lives of other families with seriously ill children better. One family facilitator noted in their journal the impact of sharing important information with families in compassionate ways is like “setting out important landmarks for the road ahead can help guide parents in the dark.”
